# Probabilistic Modeling of Dietary Arsenic Exposure and Dose and Evaluation with 2003–2004 NHANES Data

**DOI:** 10.1289/ehp.0901205

**Published:** 2009-11-23

**Authors:** Jianping Xue, Valerie Zartarian, Sheng-Wei Wang, Shi V. Liu, Panos Georgopoulos

**Affiliations:** 1 U.S. Environmental Protection Agency, Office of Research and Development, National Exposure Research Laboratory, Research Triangle Park, North Carolina, USA; 2 Graduate Institute of Environmental Health, National Taiwan University, Taipei, Taiwan; 3 Environmental and Occupational Health Sciences Institute, Piscataway, New Jersey, USA

**Keywords:** arsenic, dietary, drinking water, exposure, MENTOR, model, probabilistic, SHEDS

## Abstract

**Background:**

Dietary exposure from food to toxic inorganic arsenic (iAs) in the general U.S. population has not been well studied.

**Objectives:**

The goal of this research was to quantify dietary As exposure and analyze the major contributors to total As (tAs) and iAs. Another objective was to compare model predictions with observed data.

**Methods:**

Probabilistic exposure modeling for dietary As was conducted with the Stochastic Human Exposure and Dose Simulation–Dietary (SHEDS-Dietary) model, based on data from the National Health and Nutrition Examination Survey. The dose modeling was conducted by combining the SHEDS-Dietary model with the MENTOR-3P (Modeling ENvironment for TOtal Risk with Physiologically Based Pharmacokinetic Modeling for Populations) system. Model evaluation was conducted via comparing exposure and dose-modeling predictions against duplicate diet data and biomarker measurements, respectively, for the same individuals.

**Results:**

The mean modeled tAs exposure from food is 0.38 μg/kg/day, which is approximately 14 times higher than the mean As exposures from the drinking water. The mean iAs exposure from food is 0.05 μg/kg/day (1.96 μg/day), which is approximately two times higher than the mean iAs exposures from the drinking water. The modeled exposure and dose estimates matched well with the duplicate diet data and measured As biomarkers. The major food contributors to iAs exposure were the following: vegetables (24%); fruit juices and fruits (18%); rice (17%); beer and wine (12%); and flour, corn, and wheat (11%). Approximately 10% of tAs exposure from foods is the toxic iAs form.

**Conclusions:**

The general U.S. population may be exposed to tAs and iAs more from eating some foods than from drinking water. In addition, this model evaluation effort provides more confidence in the exposure assessment tools used.

Human exposure to arsenic (As) can occur via different routes. A well-known early medical report about As exposure and adverse health effects discussed cancer associated with dermal exposure to As-containing medication used for treating some forms of skin diseases (Hutchinson 1887). Later studies on occupational populations exposed to As compounds in industrial environments demonstrated that respiratory inhalation is a primary route of occupational As exposure, but ingestion and dermal exposure can be significant in specific situations (Occupational Safety and Health Administration 2005; World Health Organization 2004).

Compared with the simpler As chemistry and easily identified As exposure in medical and occupational fields, As chemistry and exposure routes for the general population are much more complex. General population As exposure varies according to local geochemistry, environmental pollution, living conditions, lifestyles, and activity patterns of the exposed populations. Better characterization of environmental As levels and human activity patterns is critical for accurately assessing the human exposure to As in the general population and the related health risks.

Many efforts in studying As exposure of and regulating As intake by the general population have been focused on the ingestion of As-contaminated water (Abernathy et al. 1999, 2003; Anetor et al. 2007; Chen et al. 1988a, 1988b; Chiou et al. 2001; National Research Council 2001; Tchounwou et al. 2003). This drinking water–focused As regulation also reflects a common understanding that inorganic As (iAs) is more harmful than organic As (oAs) (Tchounwou et al. 2003). A recent publication concluded that typical and high-end background exposures to iAs in the U.S. population do not present elevated risks of carcinogenicity (Boyce et al. 2008). However, other reports show significant dietary intake of iAs via food and even show food as a greater source of iAs intake than is drinking water (Meacher et al. 2002; Schoof et al. 1999a, 1999b). Yost et al. (2004) estimated dietary intake of iAs in U.S. children as 3.2 μg/day on average. A recent study shows that, in three U.S. counties, the food intake pathway is the dominant contributor to total As (tAs) exposure and dose (Georgopoulos et al. 2008).

In this study we extend findings from the previous studies by *a*) assessing the dietary tAs and iAs exposure using the peer-reviewed U.S. Environmental Protection Agency (EPA) Stochastic Human Exposure and Dose Simulation (SHEDS) model (SHEDS 2007), *b*) using more recent and larger databases representative of the U.S. population for food consumption and As concentrations in food and drinking water, and *c*) conducting model evaluation using duplicate diet and biomarker data. We used a population-based dietary exposure model, one module of the SHEDS model (SHEDS 2007; Xue et al. 2006; Zartarian et al. 2006), to estimate the exposure of As (tAs and iAs) from both food and drinking water. We linked the total predicted exposure with the Modeling ENvironment for TOtal Risk with Physiologically Based Pharmacokinetic Modeling for Populations (MENTOR-3P) system (Georgopoulos and Lioy 2006) to estimate the speciated As in urine. We compared the model results with biomarkers of tAs and As species measured in the 2003–2004 National Health and Nutrition Examination Survey (NHANES 2003–2004). Using large data sets of food consumption from NHANES, As concentrations in drinking water and various foods from the U.S. Food and Drug Administration (FDA) and Natural Resources Defense Council databases, and urinary biomarkers from NHANES (same individuals as for food consumption data), we demonstrate that dietary exposure can be a significant route for human exposure to both tAs and iAs.

## Materials and Methods

### Food consumption data

We used NHANES (2003–2004) data for model inputs regarding the amount of food and water consumed by individuals. This database contains 16,934 person-days of real-time dietary consumption data—that is, amounts of food and drinking water recorded instantly by individuals for each separate eating occasion. The average number of eating occasions is approximately 4.8 times per person per day. The U.S. EPA’s Food Consumption Intake Database (FCID) containing recipe files with 553 food commodities was applied where needed to break down NHANES food reported into raw agricultural commodities (RACs).

### tAs and iAs concentrations in food and drinking water

We used tAs residue data from the FDA’s ongoing Total Dietary Survey (TDS), also known as the market basket study (FDA 1991–2004). TDS collects and analyzes approximately 280 foods for pesticide residues, industrial chemicals, and toxic and nutrient elements. Foods collected in the TDS are prepared as “table ready,” that is, as would be consumed, for realistic estimates of dietary intake of those targeted components. As water concentrations recorded in the Natural Resources Defense Council database (Natural Resources Defense Council 2000) were used and assumed to be tAs. This database reported average and maximum As concentrations (a total of 8,970 records) in water from 25 U.S. states. The As drinking water concentration data were weighted by population and fitted for the best distribution, to yield a lognormal distribution with 1.03 ppb as the geometric mean and 4.06 ppb as the geometric standard deviation. We derived iAs concentration in each food commodity by using iAs percentage in the same food category as reported by Schoof et al. (1999a, 1999b).

### Biomarker data for As exposure

We compared urinary biomarker data from the same individuals for consumption data (2,573 records) from the NHANES with model predictions during the same time period as the consumption data were collected. Detection rates for tAs, dimethylarsinic acid (DMA), arsenobetaine, and monomethylarsonic acid (MMA) were 98.9%, 87.4%, 66.7%, and 36.2%, respectively. Because the detection rates for iAs and other species were very low (1–7%), our model evaluation study using biomarker data focused primarily on tAs.

### Models used

We used a SHEDS model developed by the U.S. EPA Office of Research and Development’s National Exposure Research Laboratory (SHEDS 2007; Xue et al. 2006; Zartarian et al. 2006) for calculating dietary and drinking water As exposures for each eating occasion of individuals, estimating the ranges of population dietary exposures, identifying key factors and contributions of food types and chemicals, and quantifying uncertainties. The exposure outputs from this SHEDS-Dietary model were used for providing input for deriving target tissue doses and biomarker levels in the population-oriented physiologically based pharmacokinetic (PBPK) modeling of MENTOR-3P developed by the Environmental and Occupational Health Sciences Institute, University of Medicine and Dentistry of New Jersey, R.W. Johnson Medical School, and Rutgers University (Georgopoulos and Lioy 2006).

### Exposure modeling

**Figure 1:**
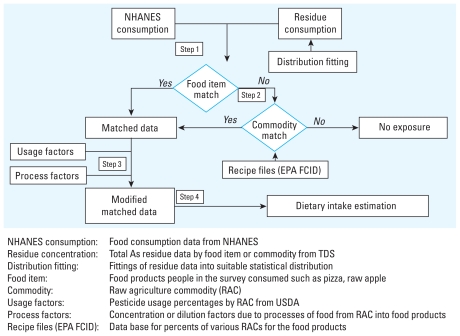
SHEDS-Dietary module overview. USDA, U.S. Department of Agriculture.

For estimating daily dietary As exposure, the detailed NHANES food diaries were used by the SHEDS-Dietary model to simulate food ingestion exposures by separate eating occasions for a simulated individual (Figure 1). The SHEDS-Dietary model can use residues for food items as consumed, as well as residues of RACs. The reported NHANES food items were matched with food items in the TDS where possible (Figure 1, step 1). If TDS residues for As were available for a particular food (e.g., rice, chicken), then SHEDS-Dietary randomly drew a TDS tAs or iAs residue from that corresponding residue distribution of the same food. Otherwise, the model applied the FCID recipe files to the NHANES food items and randomly selected a residue for each of the RAC ingredients according to the recipe (Figure 1, step 2).

Through the recipe files, the unmatched foods consumed were matched by RAC so that residues for those foods could be calculated. The SHEDS-Dietary model drew the same residue value if that RAC was found in the same foods. Assignment of residues for nondetect values depended on the commodity: if there was at least one detection, half the limit of detection was assigned; if no As values were detected, zero values were assigned. For each NHANES food diary, SHEDS-Dietary was applied using Monte Carlo simulation by selecting a residue value from an empirical distribution for each TDS food or RAC. Although a particular commodity may be used in multiple foods, the cooking method may differ, so it will have a different food form. Process factors can then be applied (Figure 1, step 3). These factors account for food changes and related concentration changes due to dilution, drying, and so on, but were not used here because of the lack of sufficient such information for our study. Each simulated individual’s exposure for each commodity was calculated by multiplying total daily consumption with corresponding residues. Aggregate daily exposure was calculated by summing exposures across all commodities:


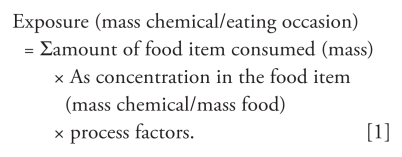


Summation of As exposures from every eating occasion for 1 day yielded the individual’s daily tAs exposure (Figure 1, step 4). In principle, both food residues and drinking water concentrations may vary by eating occasion and/or across foods consumed within an eating occasion.

For modeling drinking water As exposures, we used the NHANES data to assess the timing and amounts of direct and indirect drinking water intake within a simulated person-day. Total drinking water consumed (both direct and indirect water, from tap, bottled, and other sources) was assumed to contain the same concentration level; that is, only one concentration value was selected in the Monte Carlo simulation for each eating occasion. Water used in cooking is one example of indirect water. The modeled drinking water exposure algorithm in SHEDS-Dietary is similar to that used for food exposure (Equation 1). One residue value is randomly selected and multiplied by total water intake to obtain drinking water exposures. Although SHEDS-Dietary can be used to model longitudinal dietary exposure as well as cross-sectional exposure, we addressed only the cross-sectional exposure based on single-day data.

### PBPK modeling

**Figure 2:**
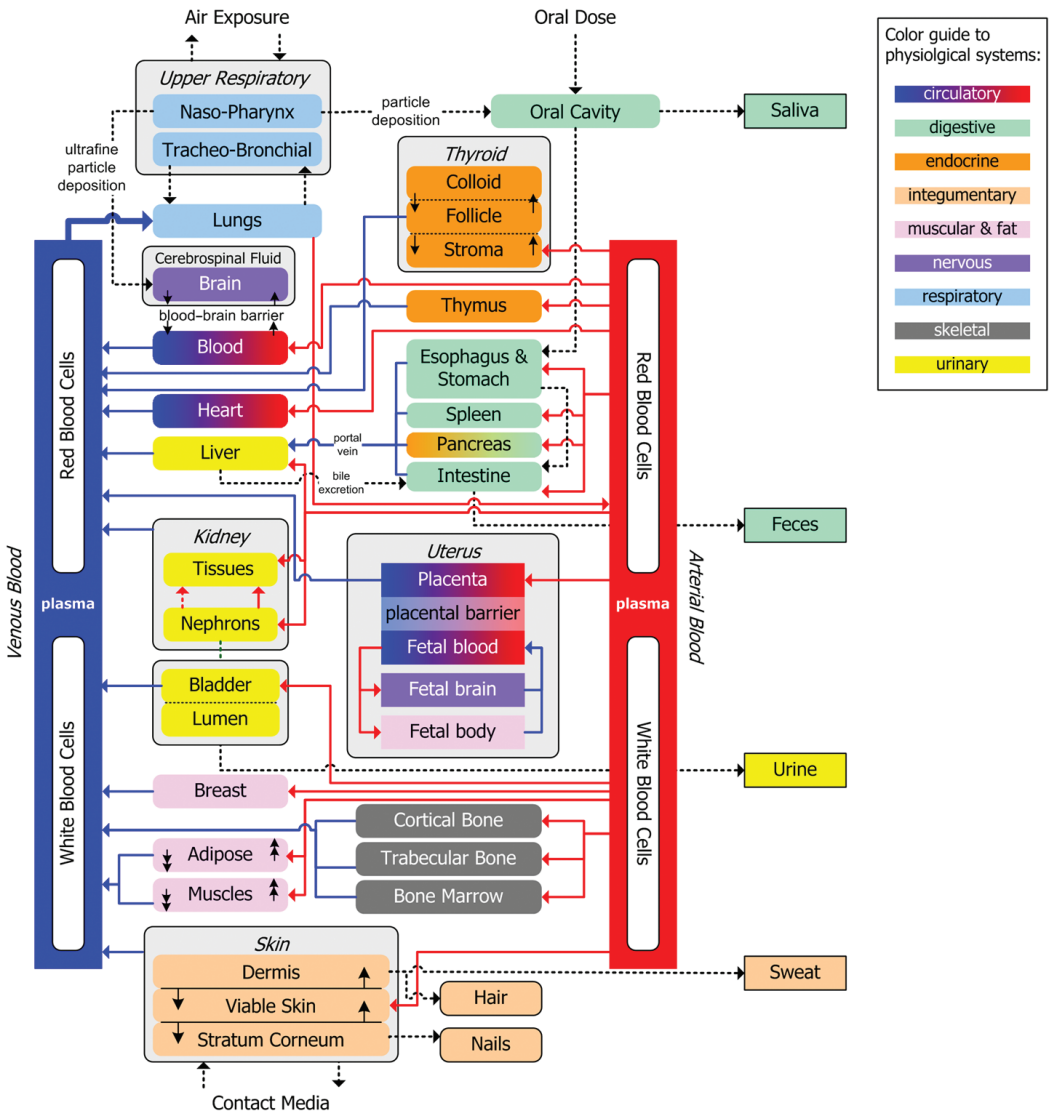
Structure of PBPK modeling of exposure to As in the MENTOR framework.

We used MENTOR-3P to represent absorption, distribution, metabolism, and excretion processes of As inside the human body by lumping together similar tissues as a set of physiologic compartments. A “flow-limited” PBPK formulation, representing a simplification of a generalized PBPK model of MENTOR-3P (Figure 2), was adopted here. This simplified PBPK model for As employed the model parameters in the work of Yu (1999a, 1999b), including fractional blood flow rates, metabolism parameters, and tissue/blood partition coefficients. The modification of calculating tissue volumes and blood flow rate based on body weight was added to this simplified population- oriented PBPK model (see Georgopoulos et al. 2008 and references therein), such that the interindividual variability of these physiologic parameters can be captured. The dynamics of four As circulating species in body compartments (arsenates, arsenites, and the As metabolites MMA and DMA) were captured using this PBPK model. Also characterized were the corresponding biomarker levels in urine.

### Model results evaluation

We conducted two types of model evaluation: *a*) SHEDS-Dietary predictions were compared with National Human Exposure Assessment Survey (NHEXAS) duplicate diet data; and *b*) linked SHEDS–MENTOR predictions were compared with NHANES biomonitoring data. Duplicate food study subjects in NHEXAS (*n* = 156) were matched by age, sex, and location with modeled results from SHEDS-Dietary (based on NHANES consumption diaries). To account for variability, we ran the model 200 times for 156 matched subjects, and selected three cumulative distribution functions according to the 5th, 50th, and 95th percentiles of the 200 simulations. Modeled estimates of tAs dose from the linked SHEDS–MENTOR predictions were compared with the NHANES urinary biomarker data for tAs. For the matched NHANES dietary consumption with NHANES biomarker data, 2,355 records were available.

## Results

Using the SHEDS-Dietary model, we calculated that the tAs exposure from food is 0.36, 1.28, and 1.40 μg/kg/day for the mean, SD, and 95th percentile, respectively, for the entire simulated population (Table 1). The tAs exposure from food for young children (≤ 5 years of age) is higher (means ranged between 0.54 and 0.62 μg/kg/day) than that shown for other age groups (means ranged between 0.25 and 0.37 μg/kg/day) (Table 1). Based on mean values in Tables 1 and 2, the tAs exposure from food predicted by SHEDS-Dietary is, on average, approximately 14 times higher than the tAs exposure from drinking water. iAs exposures from drinking water are 0.025, 0.104, and 0.107 μg/kg/day for the mean, SD, and 95th percentile, respectively (Table 2). There is no clear age group difference in the drinking water As exposure.

The iAs exposure from food for young children (≤ 5 years of age) is higher (means ranged between 0.08 and 0.23) than that shown for other age groups (means ranged between 0.03 and 0.04) (Table 1). The iAs exposure from food predicted by SHEDS-Dietary model (Table 1) is on average two times higher than the tAs exposure from drinking water (Table 2). Thus, even if we assume all As in the drinking water exists in the iAs forms, the dietary food iAs exposure by the modeled general U.S. population is still greater than the drinking water exposure. Summarizing the iAs contribution by food commodities, we estimate that about 10% of tAs exposure from foods is the toxic iAs form.

Among biomarkers analyzed for As exposure in the NHANES subjects, arsenobetaine and DMA had high concentrations, with means of 8.4 and 5.4 μg/L, respectively, whereas the mean concentration for tAs in the urine was 18.4 μg/L [see Supplemental Material, Table 4s (available online (doi:10.1289/ehp.0901205.S1 via http://dx.doi.org)].

**Figure 3:**
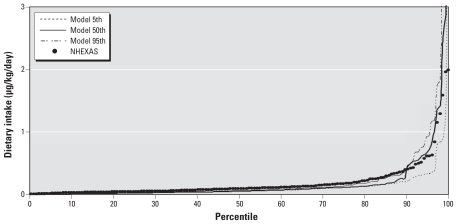
SHEDS-Dietary exposure model evaluation with NHEXAS duplicate food survey (nondetects replaced with one-half the limit of detection).

Compared with the NHEXAS duplicate diet data, our SHEDS-Dietary modeling of tAs exposure from foods performed reasonably well (Figure 3). Among 156 paired comparisons, the mean ± SD of SHEDS-Dietary estimates for tAs exposure from food was 0.192 ± 0.561 μg/kg/day, compared with 0.185 ± 0.3 shown by the NHEXAS duplicate diet analysis (Table 3).

**Figure 4:**
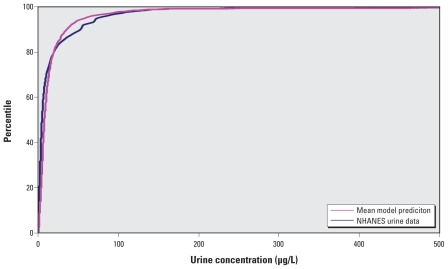
tAs model evaluation for SHEDS and MENTOR PBPK with NHANES urine data.

The linked SHEDS–MENTOR model also predicted well the tAs in urine (Figure 4). The SAS (version 9.2; SAS Insitute Inc., Cary, NC) regression analysis showed a good fit with a slope of 1.4 and *R*^2^ of 0.91 for the logarithmic-transformed predicted and measured values. The means of model predictions and NHANES urine measurements of tAs are 18.32 and 18.06 μg/L, respectively (Table 3).

**Figure 5:**
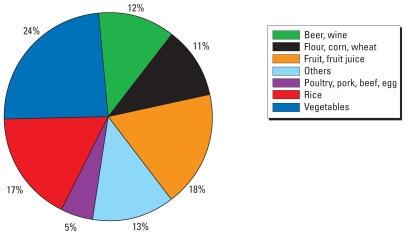
Contributions of iAs intake by foods.

The five major food contributors to tAs exposure were fish (60%), shellfish (9%), rice (7%), fruit juices and fruits (5%), and meats (5%) [see Supplemental Material, Figure 1s (doi:10.1289/ehp.0901205.S1)]. The major food contributors to iAs exposure were vegetables (24%), fruit juices and fruits (18%), rice (17%), beer and wine (12%), and flour, corn, and wheat (11%) (Figure 5).

## Discussion

It is challenging to study As exposure in the general human population because many variables affect the processes, and obtaining relevant information has numerous limitations. Unlike the study of occupational As exposure, where populations are relatively homogeneous, As compounds are easy to identify, and exposure routes are limited. As exposure in the general population is complicated with subject heterogeneity, different As species, and multiple exposure routes. Some information easily obtainable from industrial settings may be difficult or too expensive to obtain in general environmental settings. Another challenge is that As from the diet exists in many forms, most as oAs, which is much less toxic than iAs. Thus, it is important to consider the different As species in As exposure and risk analysis. Using some modeling approaches to estimate general human exposure to As and to identify some data gaps or assumption deficiencies is helpful for understanding As exposure in the general population.

Previous studies have shown that, for most people in the general population, diet may be the largest source of exposure to As (MacIntosh et al. 1996). For example, MacIntosh et al. (1997) reported that mean dietary intakes of tAs is 50.6 μg/day for females and 58.5 μg/day for males. Some recent studies suggested that dietary exposure to As may exceed the maximum As intake from drinking water in areas where elevated As levels were found in rice (Williams et al. 2007). Other studies have shown a greater intake of toxic iAs from food compared with that from drinking water (e.g., Meacher et al. 2002). Schoof et al. (1999a, 1999b) estimated that intake of iAs in the U.S. diet ranges from 1 to 20 μg/day, with a mean of 3.2 μg/day. An estimation of dietary iAs intake by U.S. children was 3.2 μg/day on average, with a range of 1.6–6.2 μg/day (Yost et al. 2004). These estimations are close to values reported in another study that showed average iAs intake ranges from 1.34 μg/day in infants to 12.54 μg/day in 60- to 65-year-olds (Tao and Bolger 1998). However, these studies of dietary As exposure are usually based on the same assumed food intake values per person, so they lack characterization of interindividual variability of exposures. Lack of data about the actual amount of food consumed accounted for at least 80% of the total uncertainty for As exposure estimation (MacIntosh et al. 1996). MacIntosh et al. (1997) also pointed out that the food consumption–food composition approach adopted in their earlier study (MacIntosh et al. 1996) did not capture all the As exposure as reflected in the empirically weighted toenail As concentration data used for validation.

In the present study we used data from NHANES, thus far the most comprehensive survey including food intakes, which has the unique advantage of containing biomarker information for the same subjects in the survey (NHANES 2003–2004). Biomarkers of exposure are independent measurements that can be used to evaluate the validity of dietary assessment methods and food composition data. Using the biomarker data from the same survey for model evaluation is more reliable, because it does not suffer from other complications such as differences between study groups related to location, lifestyle, living conditions, and other potential confounding factors.

The NHANES data are also more recent than data such as the Continuing Survey of Food Intakes by Individuals 1994–1996, 1998 (Agricultural Research Service 2009) used in previous studies. Compared with previous As exposure modeling, the SHEDS-Dietary model we used in this study performed food item matching and incorporated usage factors in the modeling. We also based the dietary intake estimation on actual eating occasions (Figure 1).

Our modeling approach yielded estimates that are very compatible with the duplicate diet data (Figure 3). The mean and 95th percentile of modeled tAs exposure (0.192 and 0.723 μg/kg/day, respectively) were very comparable to As intakes from the NHEXAS duplicate food study (0.185 and 0.612 μg/kg/day, respectively) for the same age, sex, and location. The combination of the SHEDS-Dietary model with MENTOR-3P also predicted urine tAs concentrations that compared well with biomarker monitoring data in the NHANES (slope = 1.4 and *R*^2^ = 0.91 with logarithmic-transformed data) (Figure 4). Thus, it seems that our modeling approach has overcome some previous deficiencies and yielded more reliable estimates.

Because of the low detection rates of iAs (1–7%) in the NHANES urine data, the evaluation of SHEDS–MENTOR modeling results for iAs could not be conducted. However, the Yu et al. PBPK model adapted for MENTOR-3P has been validated with experimental observations from the literature for urinary biomarker levels of speciated arsenic such as in Buchet et al. (1981), Pomroy et al. (1980), and Johnson and Farmer (1991) as described by Yu (1999a, 1999b). Because the TDS study provided only tAs concentrations in foods, we used the iAs percentage in the same food category as reported by Schoof et al. (1999a, 1999b) to derive iAs food concentrations. This assumption could result in uncertainties of estimated iAs exposure from foods, which could be carried into the subsequent PBPK modeling analysis for estimating target tissue doses and biomarker levels of iAs.

Our results in general are consistent with those reported in previous studies. For example, a duplicate diet study of children in Germany showed weekly As intake as 2.31 μg/kg body weight/week, which is equivalent to 0.33 μg/kg/day and is close to our estimate of 0.39 μg/kg/day (Wilhelm et al. 2003). These are compatible with our estimates of 7.2 and 3.5 μg/day for 1- to 2-year-olds and 10.8 and 4.1 μg/day for 3- to 5-year-olds. Another study showed that average intake of tAs for the general U.S. population estimated by the Dietary Exposure Potential Model is 0.653 μg/kg/day (Moschandreas et al. 2002), which is similar to our result of 0.39 μg/kg/day for the same population. Even when iAs is specifically considered, our results are also within the wider range of iAs exposures reported in previous such studies. For example, Schoof et al. (1999a, 1999b) estimated the iAs intake from U.S. diet to be 1–20 μg/day with a mean of 3.2 μg/day, and Tao and Bolger (1998) reported it as 1.34 μg/day in infants and 12.54 μg/day in adults 60–65 years of age. Our results of the major food contributors to As exposure are consistent with the As levels measured in various foods in U.S. markets (Tao and Bolger 1998).

Our modeling assessment advances the science by using the large and recent databases from NHANES, TDS, NHEXAS, and the Natural Resources Defense Council to estimate As intake for the U.S. general population from food and drinking water. Other unique aspects of research presented in this article are evaluation of tAs intake estimates using duplicate food survey data from NHEXAS, and using urine biomarker data from NHANES to evaluate the SHEDS–MENTOR model predictions. The integrated exposure and dose modeling application presented in this article for As has not been attempted before for a large general population (e.g., the U.S. general population), to our knowledge, in the exposure-related literature. The SHEDS-Dietary model and the linked SHEDS-Dietary–MENTOR-3P model predictions compared well with the measured duplicate diet data and urine biomarker data, respectively; thus, this was an important model evaluation effort to provide more confidence in these predictive exposure assessment tools.

## Conclusions

The relationship between As intake from drinking water and related health effects has been well studied previously. Using rich data sets and state-of-the-science models, we found that the general U.S. population may be exposed to tAs and toxic iAs through the dietary route more from eating some As-containing foods than from drinking As-containing water. The major food contributors to tAs exposure were fish, shellfish, rice, fruit juices and fruits, and meats; the major food contributors to iAs exposure were vegetables, fruit juices and fruits, rice, beer and wine, and flour, corn, and wheat. Approximately 10% of tAs exposure from foods is the toxic iAs form.

Our study reinforces and expands on previous observations that dietary As exposure via food is an important route for As intake by the general population and that in some cases it can be even a greater source of As exposure than drinking water. Thus, for complete exposure analysis and risk assessment in the general population, iAs intake from food should be considered in addition to iAs intake from drinking water.

## Figures and Tables

**Table 1:** SHEDS modeled As exposure from food using NHANES (2003–2004) data (μg/kg/day).

As species/ age group (years)	*n*	Mean ± SD	Percentile					
			5th	25th	50th	75th	95th	99th
tAs								
0 to < 1	757	0.62 ± 0.53	0.05	0.27	0.56	0.84	1.45	2.08
1–2	1,068	0.54 ± 1.23	0.05	0.13	0.26	0.46	2.06	5.06
3–5	953	0.54 ± 1.74	0.03	0.11	0.2	0.36	2.2	5.6
6–12	2,190	0.37 ± 1.69	0.02	0.05	0.1	0.22	1.24	4.28
13–19	3,576	0.25 ± 1.04	0.01	0.03	0.06	0.14	1.02	3.58
20–49	4,221	0.33 ± 1.09	0.01	0.03	0.07	0.17	1.41	4.12
≥ 50	3,804	0.32 ± 1.2	0.01	0.03	0.05	0.15	1.35	4.91
All ages	16,931	0.36 ± 1.28	0.01	0.04	0.08	0.24	1.4	4.45

iAs								
0 to < 1	757	0.23 ± 0.19	0.01	0.09	0.21	0.31	0.53	0.8
1–2	1,068	0.1 ± 0.12	0.01	0.04	0.07	0.12	0.29	0.59
3–5	953	0.08 ± 0.11	0.01	0.03	0.05	0.09	0.21	0.4
6–12	2,190	0.04 ± 0.06	0	0.01	0.03	0.05	0.13	0.25
13–19	3,576	0.03 ± 0.05	0	0.01	0.01	0.03	0.09	0.21
20–49	4,221	0.03 ± 0.07	0	0.01	0.02	0.04	0.11	0.28
≥ 50	3,804	0.03 ± 0.07	0	0.01	0.01	0.03	0.09	0.22
All ages	16,931	0.05 ± 0.09	0	0.01	0.02	0.05	0.19	0.41

**Table 2:** SHEDS modeled As exposure from drinking water (μg/kg/day).

			Percentile					
Age group (years)	*n*	Mean ± SD	5th	25th	50th	75th	95th	99th
0 to < 1	756	0.014 ± 0.083	0.000	0.000	0.000	0.000	0.053	0.412
1–2	1,064	0.031 ± 0.108	0.000	0.000	0.002	0.019	0.150	0.397
3–5	944	0.036 ± 0.150	0.000	0.000	0.004	0.021	0.152	0.539
6–12	2,179	0.030 ± 0.156	0.000	0.000	0.003	0.016	0.108	0.441
13–19	3,566	0.019 ± 0.092	0.000	0.000	0.002	0.011	0.076	0.281
20–49	4,218	0.026 ± 0.087	0.000	0.000	0.002	0.016	0.113	0.414
≥ 50	3,797	0.025 ± 0.084	0.000	0.000	0.004	0.019	0.107	0.344
All ages	16,883	0.025 ± 0.104	0.000	0.000	0.002	0.016	0.107	0.374

**Table 3:** Comparison of tAs intake and urinary excretion with SHEDS results and the PBPK model.

			Percentile			
Data source	*n*	Mean ± SD	25th	50th	75th	95th
Comparison of tAs intake (μg/kg/day) of NHEXAS duplicates and SHEDS results						
NHEXAS	156	0.185 ± 0.3	0.049	0.095	0.174	0.612
SHEDS	156	0.192 ± 0.561	0.024	0.052	0.115	0.723

Comparison of tAs in urine (μg/L) from NHANES data and PBPK model						
PBPK model	2,355	18.32 ± 46.86	4.7	8.1	16.1	
Measured concentration	2,355	18.06 ± 42.12	2.5	4.89	14.64	74.84
